# IOA-244 is a Non–ATP-competitive, Highly Selective, Tolerable PI3K Delta Inhibitor That Targets Solid Tumors and Breaks Immune Tolerance

**DOI:** 10.1158/2767-9764.CRC-22-0477

**Published:** 2023-04-14

**Authors:** Zoë Johnson, Chiara Tarantelli, Elisa Civanelli, Luciano Cascione, Filippo Spriano, Amy Fraser, Pritom Shah, Tyzoon Nomanbhoy, Sara Napoli, Andrea Rinaldi, Karolina Niewola-Staszkowska, Michael Lahn, Dominique Perrin, Mathias Wenes, Denis Migliorini, Francesco Bertoni, Lars van der Veen, Giusy Di Conza

**Affiliations:** 1 iOnctura SA, Geneva, Switzerland.; 2 Institute of Oncology Research, Faculty of Biomedical Sciences, USI, Bellinzona, Switzerland.; 3 SIB, Swiss Institute of Bioinformatics, Lausanne, Switzerland.; 4 Cancer Research Horizons, Jonas Webb Building, Cambridge, United Kingdom.; 5 ActivX Biosciences, Inc., La Jolla, California.; 6 Merck Healthcare KGaA, Darmstadt, Germany.; 7 Brain Tumor and Immune Cell Engineering Group, Faculty of Medicine, University of Geneva, Geneva, Switzerland.; 8 Center for Translational Research in Onco-Hematology, University of Geneva, Geneva, Switzerland.; 9 Swiss Cancer Center Leman, Geneva and Lausanne, Switzerland.; 10 Department of Oncology, Geneva University Hospitals (HUG), Geneva, Switzerland.; 11 Oncology Institute of Southern Switzerland, Ente Ospedaliero Cantonale, Bellinzona, Switzerland.

## Abstract

PI3K delta (PI3Kδ) inhibitors are used to treat lymphomas but safety concerns and limited target selectivity curbed their clinical usefulness. PI3Kδ inhibition in solid tumors has recently emerged as a potential novel anticancer therapy through the modulation of T-cell responses and direct antitumor activity. Here we report the exploration of IOA-244/MSC2360844, a first-in-class non–ATP-competitive PI3Kδ inhibitor, for the treatment of solid tumors. We confirm IOA-244’s selectivity as tested against a large set of kinases, enzymes, and receptors. IOA-244 inhibits the *in vitro* growth of lymphoma cells and its activity correlates with the expression levels of *PIK3CD*, suggesting cancer cell–intrinsic effects of IOA-244. Importantly, IOA-244 inhibits regulatory T cell proliferation while having limited antiproliferative effects on conventional CD4^+^ T cells and no effect on CD8^+^ T cells. Instead, treatment of CD8 T cells with IOA-244 during activation, favors the differentiation of memory-like, long-lived CD8, known to have increased antitumor capacity. These data highlight immune-modulatory properties that can be exploited in solid tumors. In CT26 colorectal and Lewis lung carcinoma lung cancer models, IOA-244 sensitized the tumors to anti-PD-1 (programmed cell death protein 1) treatment, with similar activity in the Pan-02 pancreatic and A20 lymphoma syngeneic mouse models. IOA-244 reshaped the balance of tumor-infiltrating cells, favoring infiltration of CD8 and natural killer cells, while decreasing suppressive immune cells. IOA-244 presented no detectable safety concerns in animal studies and is currently in clinical phase Ib/II investigation in solid and hematologic tumors.

**Significance:**

IOA-244 is a first-in-class non–ATP-competitive, PI3Kδ inhibitor with direct antitumor *in vitro* activity correlated with PI3Kδ expression. The ability to modulate T cells, *in vivo* antitumor activity in various models with limited toxicity in animal studies provides the rationale for the ongoing trials in patients with solid tumors and hematologic cancers.

## Introduction

PI3K delta (PI3Kδ, or p110δ) is a class IA member of the phosphatidylinositol (PI) kinase family that is predominantly expressed in immune cells, where it associates with other PI3K regulatory subunits and mediates activation signals (1). The enzyme is essential for survival and proliferation of B-cell lymphomas (2–5), and PI3Kδ inhibitors with different degrees of isoform selectivity have been developed to treat relapsed or refractory indolent lymphomas, for example, follicular lymphoma (FL), or chronic lymphocytic leukemia (CLL; refs. 6, 7). Although these inhibitors have shown durable clinical responses, they are also associated with variable degrees of toxicity, ultimately leading to withdrawals of the accelerated approvals received for these compounds in FL (8).

PI3Kδ inhibitors are also applicable to solid tumors, in which they act directly on the tumor cells or by breaking immune cell–mediated tolerance to cancer (9–13). Regulatory T cells (T_reg_) and myeloid-derived suppressive cells (MDSC) are suppressive immune cells that inhibit CD8 T-cell activation and antitumor function. Direct PI3Kδ inactivation in T_reg_ and MDSCs leads to an increase in effector T cell (T_eff_) activity, which in turn results in tumor regression (9, 11, 14). Furthermore, PI3Kδ inhibition plays an important role in the recruitment of macrophages to tumor sites in breast cancer models (15), and tumor immunosuppression can be partly mediated by B cells and an altered cytokine milieu (16). Indeed, data from a phase Ib study show an increased intratumor CD8^+^:T_reg_ ratio and reduced number of intratumor T_reg_ in patients with advanced solid tumors treated with the combination of a PI3Kδ with a PD-1 (programmed cell death protein 1) mAb (17). Moreover, PI3Kδ is overexpressed in several solid tumors (15, 18), and its pharmacologic inhibition exerts antitumor activity in various models (15, 19, 20). PI3Kδ expression appears to increase during the progression of breast and liver cancer, and preclinical tumor models with high PI3Kδ expression levels are sensitive to selective PI3Kδ inhibitors (15, 19). Unfortunately, so far the administration of PI3Kδ inhibitors in patients with solid tumors has been associated with rapid onset of toxicities, which are even more aggravated than in patients with hematologic cancers (12, 13). Hence, intermittent dosing or lower doses have been suggested to reduce the risk of toxicity in patients receiving current PI3Kδ inhibitors (13).

The first non–ATP-competitive PI3Kδ inhibitor IOA-244 (MSC2360844) was originally developed to treat autoimmune diseases, in which it shows efficacy in preclinical models of systemic lupus erythematosus and lupus nephritis (21). Here, we describe the use of IOA-244 for cancer treatment and we show preclinical data of efficacy in different tumor models, accompanied by a remodeling of the tumor microenvironment, sustaining the ongoing phase I study in patients with solid tumors and hematologic cancers (NCT04328844; refs. 22, 23). Importantly, IOA-244 shows high selectivity and low toxicity both *in vitro* and *in vivo.*

## Materials and Methods

### *In Vitro* Activity Assay

The activity of compounds was measured in a scintillation proximity assay (SPA) format using PI as a substrate in micellar form and phosphatidyl-L-serine as a lipid carrier as described by Haselmayer and colleagues (1)

### *In Vitro* Selectivity Assay

Selectivity in Jurkat cell lysate was measured using the KiNativ method. Jurkat cells were lysed by sonication in lysis buffer [50 mmol/L HEPES, pH 7.5, 150 mmol/L NaCl, 0.1% Triton-X-100, phosphatase inhibitors (Cocktail II AG Scientific #P-1518)]. After lysis, the samples were cleared by centrifugation, and the supernatant collected for probe labeling. Final protein concentration of lysates was 5 mg/mL. A total of 5 μL compound was added from a 100X stock solution in DMSO to 445 μL of lysate in duplicate. A total of 5 μL of DMSO was added to 445 μL of lysate in quadruplicate for controls. After 15 minutes incubation, 50 μL of a 10× aqueous solution of the desthiobiotin-adenosine triphosphate was added to each sample for a final probe concentration of 20 μmol/L and incubated with the samples for 15 minutes. The final DMSO concentration was 1%. Following the probe reaction, samples were prepared for targeted mass spectrometry (MS) analysis using the ActivX standard protocol (24).

### Human Whole Blood Selectivity Assay

Fresh human blood was aliquoted into 50 mL centrifuge tubes. Compound was added from a 1,000× DMSO stock solution of the desired final concentration. For control cells (no compound), an equivalent volume of DMSO was added. Cells were incubated at 37°C for 1 hour. After 1 hour, the peripheral blood mononuclear cell fraction was isolated using Leucosep (Greiner Bio-One) tubes using the manufacturers’ protocol, and the cell pellets stored at −80°C until needed for processing. Cells were lysed by sonication in lysis buffer [50 mmol/L HEPES, pH 7.5, 150 mmol/L NaCl, 0.1% Triton-X-100, phosphatase inhibitors (Cocktail II AG Scientific #P-1518)]. The ratio of the volume of lysis buffer to cell pellet was kept at 10:1. After lysis, the samples were cleared by centrifugation, and the supernatant collected for probe labeling. A total of 50 μL of a 10x aqueous solution of the ATP probe is added to 450 μL each sample (final concentration of probe was 20 μmol/L). All samples were then incubated for 15 minutes. Following the probe reaction, samples were prepared for targeted MS analysis using the ActivX standard protocol (24).

### Cell Lines

Cell line identities were validated with the Promega GenePrint 10 System kit, and all experiments with the cells were performed within 1 month of their being thawed. Cells were periodically tested to confirm *Mycoplasma* negativity using the MycoAlert *Mycoplasma* Detection Kit (Lonza), at least 2 weeks before performing experiments. Cells were subcultured every 3 days. Origin and RRDI of the lymphoma cell lines are listed in Table 1.

**TABLE 1:** Origin and RRDI of the lymphoma cell lines

B T	Histo	Cell_line	From	RRDI
B	murine	A20	ATCC	CVCL_1940
B	HL	AM-HLH	Dr Hitoshi Ohno (Tenri Hospital, Japan)	CVCL_IP72
B	GCB DLBL	DB	ATCC	CVCL_1168
T	NK lymphoma	DERL7	Satu Mustjoki (Helsinki, Finland)	CVCL_2017
B	GCB DLBL	DOHH2	Finbarr Cotter (London, United Kongdom)	CVCL_1179
B	MZL	ESKOL	Brunangelo Falini (Perugia, Italy)	CVCL_B398
B	GCB DLBL	FARAGE	ATCC	CVCL_3302
T	PTCL-NOS	FEPD	Laurence Lamant (Toulouse, France)	CVCL_H614
B	MCL	GRANTA519	Deutsche Sammlung von Mikro-organismen und Zellkulturen (DSMZ)	CVCL_1818
T	SS	H9	ATCC	CVCL_1240
B	MZL	HAIR-M	Brunangelo Falini (Perugia, Italy)	CVCL_B401
B	ABC DLBCL	HBL1	Fabio Martinon (Lausanne, CH)	CVCL_M572
B	MZL	HC-1	Brunangelo Falini (Perugia, Italy)	CVCL_1243
B	CLL	HG3	Davide Rossi (Bellinzona, Switzerland)	CVCL_Y547
T	CTCL	HH	ATCC	CVCL_1280
T	SS	HUT78	ATCC	CVCL_0337
B	MCL	JEKO1	Eisaku Kondo, (Okayama, Japan)	CVCL_1865
B	MCL	JVM2	Elias Campo (Barcelona, Spain)	CVCL_1319
B	PMBCL	KARPAS-1106P	Sigma-Aldrich ora Merck (St. Louis, USA)	CVCL_1821
B	SMZL	KARPAS-1718	José Ángel Martinez-Climent (Pamplona, Spain)	CVCL_2539
T	ALCL, ALK+	KARPAS-299	Giorgio Inghirami (New York, NY)	CVCL_1324
B	GCB DLBL	KARPAS-422	Deutsche Sammlung von Mikro-organismen und Zellkulturen (DSMZ)	CVCL_1325
T	NK lymphoma	KHYG	Satu Mustjoki (Helsinki, Finland)	CVCL_2976
T	ALCL, ALK+	KIJK	Giorgio Inghirami (New York, NY)	CVCL_2093
B	HL	KM-H2	Ralf Kuppers (Essen, DE)	CVCL_1330
B	HL	L-1236	Ralf Kuppers (Essen, DE)	CVCL_2096
B	HL	L428	Ralf Kuppers (Essen, DE)	CVCL_1361
T	ALCL, ALK+	L82	Giorgio Inghirami (New York, NY)	CVCL_2098
T	cALCL, ALK-	MAC1	Giorgio Inghirami (New York, NY)	CVCL_H631
B	MCL	MAVER1	Alberto Zamò (Verona, Italy)	CVCL_1831
B	CLL	MEC1	Alberto Zamò (Verona, Italy)	CVCL_VR92
B	MCL	MINO	Robert Kridel (Vancouver, Canada)	CVCL_UW35
T	CTCL	MJ	ATCC	CVCL_1414
T	NK lymphoma	NK92	Satu Mustjoki (Helsinki, Finland)	CVCL_2142
T	NK lymphoma	NKYS	Satu Mustjoki (Helsinki, Finland)	CVCL_8461
B	GCB DLBL	OCILY1	Laura Pasqualucci (New York, NY)	CVCL_1879
B	ABC DLBCL	OCILY10	Laura Pasqualucci (New York, NY)	CVCL_8795
B	GCB DLBL	OCILY18	Laura Pasqualucci (New York, NY)	CVCL_1880
B	GCB DLBL	OCILY19	Louis M. Staudt (Bethesda, MD)	CVCL_1878
B	ABC DLBCL	OCILY3	Laura Pasqualucci (New York, NY)	CVCL_8800
B	GCB DLBL	OCILY7	Laura Pasqualucci (New York, NY)	CVCL_1881
B	GCB DLBL	OCILY8	Laura Pasqualucci (New York, NY)	CVCL_8803
B	CLL	PCL12	Cristina Scielzo (Milan, Italy)	CVCL_2H32
B	GCB DLBL	PFEIFFER	ATCC	CVCL_3326
B	ABC DLBCL	RCK8	Laura Pasqualucci (New York, NY)	CVCL_1883
B	MCL	REC1	Finbarr Cotter (London, United Kingdom)	CVCL_1884
B	ABC DLBCL	RI1	Laura Pasqualucci (New York, NY)	CVCL_1885
B	MCL	SP49	Robert Kridel (Vancouver, Canada)	CVCL_D022
B	MCL	SP53	Robert Kridel (Vancouver, Canada)	CVCL_C122
B	SMZL	SSK41	José Ángel Martinez-Climent (Pamplona, Spain)	CVCL_C123
T	ALCL, ALK+	SUDHL1	Giorgio Inghirami (New York, NY)	CVCL_0538
B	GCB DLBL	SUDHL10	Fabio Martinon (Lausanne, CH)	CVCL_1889
B	GCB DLBL	SUDHL16	Laura Pasqualucci (New York, NY)	CVCL_1890
B	ABC DLBCL	SUDHL2	Laura Pasqualucci (New York, NY)	CVCL_9550
B	GCB DLBL	SUDHL4	Laura Pasqualucci (New York, NY)	CVCL_0539
B	GCB DLBL	SUDHL5	Laura Pasqualucci (New York, NY)	CVCL_1735
B	GCB DLBL	SUDHL6	Louis M. Staudt (Bethesda, MD)	CVCL_2206
B	GCB DLBL	SUDHL8	Laura Pasqualucci (New York, NY)	CVCL_2207
B	ABC DLBCL	TMD8	Fabio Martinon (Lausanne, CH)	CVCL_A442
B	GCB DLBL	TOLEDO	Miguel A. Piris (Santander, Spain)	CVCL_3611
B	ABC DLBCL	U2932	Bettina Borisch (Geneva, CH)	CVCL_1896
B	MCL	UPN1	Robert Kridel (Vancouver, Canada)	CVCL_A795
B	GCB DLBL	VAL	José Ángel Martinez-Climent (Pamplona, Spain)	CVCL_1819
B	SMZL	VL51	José Ángel Martinez-Climent (Pamplona, Spain)	CVCL_3169
B	GCB DLBL	WSUDLCL2	Stefano Casola (Milan, Italy)	CVCL_1902
T	NK lymphoma	YT	Satu Mustjoki (Helsinki, Finland)	CVCL_1797
B	MCL	Z138	Robert Kridel (Vancouver, Canada)	CVCL_B077

### Cellular Proliferation Assay

Cells were diluted in the corresponding ATCC recommended medium and dispensed in a 384-well plate, depending on the cell line used, at a density of 200 to 6,400 cells per well in 45 μL medium. For each used cell line, the optimal cell density is used. The margins of the plate were filled with PBS. Plated cells were incubated in a humidified atmosphere of 5% CO_2_ at 37°C. After 24 hours, 5 μL of compound diluted in DMSO was added and plates were further incubated. The final DMSO concentration during incubation was 0.4% in all wells. At *t* = end, 24 μL of ATPlite 1Step (PerkinElmer) solution was added to each well, and subsequently shaken for 2 minutes. After 10 minutes of incubation in the dark, the luminescence was recorded on an Envision multimode reader (PerkinElmer). The cellular doubling times of all cell lines were calculated from the *t* = 0 hours and *t* = end growth signals of the untreated cells. The GI_50_ (the concentration of drug at which 50% growth inhibition is achieved) is associated with the luminescence signal and was calculated by: ((luminescence_untreated,_*_t_*_= end_ – luminescence*_t_*_= 0_) /2) + luminescence*_t_*_= 0_.

### Screening of IOA-244 in Lymphoma

#### Assessment of Antiproliferative Activity

Lymphoma cell proliferation was assessed as described previously (25, 26). Lymphoma cell lines were manually seeded in 96-well plate at the concentration of 50,000 cells/mL (10,000 cells in each well). Treatment titration with increasing doses of IOA-244 (13.7 nmol/L to 10 μmol/L; 1- to 3-fold dilution) was performed using Tecan D300e Digital Dispenser (Tecan). After 72 hours, viable cells were determined using MTT [3-(4,5-dimethylthiazolyl-2)-2, 5-diphenyltetrazoliumbromide] and the reaction stopped after 4 hours with SDS. The day after absorbance is read at 570 nm using the Cytation 3 instrument (BioTek). AUC values were calculated from dose–response curves with the percentage of proliferating cells on the *Y*-axis and drug concentrations on the *X*-axis, using the Prism software v8.0 (GraphPad Software).

#### Transcriptome Profiling

For each cell line, 5 millions of cells in exponential growth were collected and resuspended in 1 mL of TRI Reagent (Sigma-Aldrich) for cell lysis. Extraction was performed by MonarchTotal RNA Miniprep kit (New England Biolabs) according to manufacturer extraction to separate RNA molecules shorter and longer than 200 nucleotides in two different fractions. Genomic DNA was digested at the initial step of extraction. Only RNA longer than 200 nucleotides was used for library preparation, after quality check by Agilent BioAnalyzer (Agilent Technologies) using the RNA 6000 Nano kit (Agilent Technologies) and concentration was determined by the Invitrogen Qubit (Thermo Fisher Scientific) using the RNA BR reagents (Thermo Fisher Scientific). The TruSeq RNA Sample Prep Kit v2 for Illumina (Illumina) was used for cDNA synthesis and addition of barcode sequences. The sequencing of the libraries was performed via a paired end run on a NextSeq500 Illumina sequencer (Illumina). At least 50 millions of reads were collected per each sample. The RNA sequencing (RNA-seq) reads quality was evaluated with FastQC (v0.11.5) and removed low-quality reads/bases and adaptor sequences using Trimmomatic (v0.35). The trimmed high-quality sequencing reads were aligned using STAR, a spliced read aligner which allows for sequencing reads to span multiple exons. On average, 85% of the sequencing reads were aligned for each sample to the reference genome (HG38). The HTSeq-count software package was then used for quantification of gene expression with GENCODE v22 as gene annotation. Data were subsetted to genes that had a counts-per-million value greater than five in at least one cell line. The data were normalized using the “TMM” method from the edgeR package and transformed to log_2_ counts-per-million using the edgeR function “cpm”. Expression values are available at the NCBI Gene Expression Omnibus (GEO; http://www.ncbi.nlm.nih.gov/geo) database. *PIK3CD* expression values were also extracted from the data (GSE94669) previously obtained using a targeted RNA-seq approach, the HTG EdgeSeq Oncology Biomarker panel (HTG Molecular Diagnostics, Inc.; ref. 26). Normalized *PIK3CD* expression values were correlated with IOA-244 drug activity, quantified as AUC, by Spearman correlation.

### Human T-cell Culture

Heparinized peripheral blood from healthy male or female volunteers was provided by Transfusion Interrégional CRS (anonymized). Donors provided written informed consent. T cells were enriched by using RosetteSep (Stem Cell Technologies) according to manufacturer's instructions. T cells (5 × 10^5^ cells/500 μL) were seeded in 48-well plates in RPMI1640 supplemented with 10% FBS, 1% penicillin/streptomycin, 1% non-essential amino acids, 1 mmol/L Na-Pyruvate, 1 mmol/L HEPES (all Gibco) and 30 IU/mL rhIL2 (Peprotech) and activated with Dynabeads Human T-Activator CD3/CD28 (Thermo Fisher Scientific) at a 1:1 cell:bead ratio in the presence of either different concentrations of IOA-244 or 200 nmol/L CAL-101. As negative control, the T cells were cultured in the solvent, DMSO (Sigma-Aldrich). After 3 days, the medium was doubled, by using the above-described medium containing freshly diluted small-molecule inhibitors and 60 IU/mL rhIL2. After 5 days of activation, the beads were removed and the T cells were then maintained at a concentration of 0.75 × 10^6^ cells/mL throughout the culture period by cell enumeration every 2–3 days, adding new medium containing freshly diluted small-molecule inhibitors and IL2. Cells were characterized by flow cytometry after 9 days of activation, with the following antibodies: anti-human CD8 – BV421 BioLegend RRID: AB_2629583, anti-human CD4 – AF532 Thermo Fisher Scientific RRID: AB_11218891, anti-human CD45RO – BUV805 BD RRID: AB_2872786, anti-human CD62 L – PerCP-Cy5.5 BioLegend RRID: AB_893396, anti-human CD127-PE BD RRID: AB_2296056, Anti-human CD25-APC RRID: AB_398598, LIVE/DEAD Fixable Yellow Dead Cell Stain Kit, for 405 nm excitation Thermo Fisher Scientific Invitrogen L34968.

### Microsomal Metabolic Stability

Pooled human liver microsomes (final protein concentration 0.5 mg/mL), 0.1 mol/L phosphate buffer pH 7.4 and test compound (final substrate concentration 1 μmol/L; final DMSO concentration 0.25%) were preincubated at 37°C prior to the addition of NADPH (final concentration 1 mmol/L) to initiate the reaction. A minus cofactor control incubation was included for each compound tested where 0.1 mol/L phosphate buffer pH 7.4 is added instead of NADPH (minus NADPH). Two control compounds were included with each species. All incubations were performed singularly for each test compound.

Each compound was incubated for 0, 5, 15, 30, and 45 minutes. The control (minus NADPH) was incubated for 45 minutes only. The reactions were stopped by transferring incubate into acetonitrile at the appropriate timepoints, in a 1:3 ratio. The termination plates were centrifuged at 3,000 rpm for 20 minutes at 4°C to precipitate the protein.

Following protein precipitation, the sample supernatants were combined in cassettes of up to four compounds, internal standard was added, and samples analyzed using generic LC/MS-MS conditions.

The gradient of the line was determined from a plot of in peak area ratio (compound peak area/internal standard peak area) against time. Subsequently, half-life and intrinsic clearance were calculated using the equations below:

Elimination rate constant (*k*) = (− gradient)
Half-life (*t*½)(min) = 0.693*k*
Intrinsic clearance (CLint)(μL/minute/mg protein) = *V* × 0.693*t*½
where *V* = Incubation volume (μL)/Microsomal protein (mg)
Dextromethorphan and verapamil were used as control compounds.

### Reactive Metabolite Formation

The formation of reactive metabolites of IOA-244 was investigated by glutathione (GSH) adduct trapping in the presence of human liver microsomes fortified with NADPH. IOA-244 (50 μmol/L) was incubated with 5 mmol/L glutathione in the presence of human liver microsomes (37°C, pH 7.4), that is, with metabolic activation. After 1 hour of incubation, further sample preparation was performed by solid phase extraction using RP-18 columns. A total of 10 μL of the purified sample material were injected into the LC/MS-MS system for analysis.

Qualitative measurements were carried out using the neutral loss scan (positive ionization mode), precursor ion scan (negative ionization mode), and product ion scan (positive ionization mode). No specific signals were detected in the IOA-244 incubations. In control incubations containing the positive control clozapine, the expected signals for putative GSH adducts were identified in the neutral loss and precursor ion scan, confirming that the conditions were appropriate to detect GSH conjugates of drugs forming electrophilic intermediates.

### Animal Studies

The animals were housed in individual ventilated cages (up to 5 mice per cage) under the following conditions:

Temperature: 20°C–26°C
Humidity: 40%–70%
Light cycle: 7:00 am to 19:00 pm light and 19:00 pm to 7:00 am (next day) darkness
Polysulfone IVC cage: size of 325 mm × 210 mm × 180 mm
Bedding material: corn cob
Diet: Mouse diet, Co^60^ irradiation sterilized dry granule food. Animals had free access during the entire study period.
Water: Reverse osmosis (RO) water, autoclaved before using. Animals had free access to sterile drinking water.
Cage identification label: number of animals, sex, strain, receiving date, treatment, study number, group number, and the starting date of the treatment, etc.
Animal identification: Animals were marked by ear coding (notch)
Adapt housing: the animals were adapted in the facility for at least 3 days.
All animal experiments have been performed according to protocol licenses approved by Institutional Review Board.

#### CT26 Syngeneic Model

CT26 tumor cells (RRID:CVCL_7254) were injected into female BALB/c mice subcutaneously at the upper back region. Treatments started when the mean tumor size reached 100–150 mm^3^. Mice were randomized according to tumor volume and assigned a group. Treatments with vehicle (1% methylcellulose), IOA-244 (30 mg/mL, twice a day, orally) and anti-PD-L1 (10 mg/kg, twice a week, i.p.; BioXcell, clone 10F.9G2, RRID:AB_2934050) were initiated after grouping. Tumor size was measured three times a week in two dimensions using a caliper, and the volume was expressed in mm^3^. At day 20, 6 mice from each group were culled and subcutaneous tumors were dissected out and weighed. Tumor samples were disaggregated according to standard protocols. The resulting cells were counted (total and viable) and stained with two panels of antibodies (i) CD45, CD3, CD4, CD8, and a viability dye; (ii) CD11b, Ly6C, Ly6G, F4/80, NKp46, CD45, CD49b, CD3, in combination with a viability marker to examine the composition of the immune infiltrate and analyzed using flow cytometry using standard protocols.

#### Lewis Lung Carcinoma, Pan02, and A20 Syngeneic Models

Lewis lung carcinoma (LLC; from SIBS, Shanghai Institutes for Biological Sciences, RRID:CVCL_4358), Pan02 (RRID:CVCL_D627), and A20 (RRID:CVCL_1940) tumor cells were thawed and cultured according to manufacturer's protocol. Cells were washed in PBS, counted, and resuspended in cold serum-free RPMI. The cells were injected subcutaneously into the rear right and left flank of female C57BL/6 mice. Mice were randomized according to tumor volume and assigned a group using the StudyDirector software (Studylog Systems, Inc.). Treatments with vehicle (1% methylcellulose), IOA-244 (30 mg/mL, twice a day, orally) and anti-PD-1 (10 mg/kg, twice a week, i.p.; BioXcell, clone RMP1-14, RRID:AB_2927529) were initiated after grouping. tumor size was measured three times a week in two dimensions using a caliper, and the volume was expressed in mm^3^.

In another A20 syngeneic experiment, the tumors were established by injecting A20 murine lymphoma cells (5 × 10^6^ cells/mouse, 100 μL of PBS) into the left flanks of female BALB/c mice. Mice were treated with vehicle (1% methylcellulose) by oral injection or with 30 mg/kg of IOA-244 twice a day five consecutive times a week (5 days on, 2 days off). Treatments started once tumor volume reaches approximately 60 mm^3^. Tumor size was measured three times per week using a digital caliper and animal body weight has been measured three times per week throughout the study. During housing and treatments, the animal status was carefully evaluated by measuring cumulative condition scores. Mice were sacrificed once tumor volume reached 2,000 mm^3^ and/or when different parameters were scored with high severity degree.

#### mPA6115-luc Murine Pancreatic Cancer Syngeneic Model

mPA6115-luc tumor cells [1 × 10^6^; luciferase expressing Kras (G12D)/Trp53 null/Pdx1-cre (KPC) MuPrime mouse tumor homograft model, Crown Biosciences] in 50 μL PBS with Matrigel (1:1) were orthotopically injected into the subcapsular region of the pancreas of female C57BL/6 mice. The randomization started 4 days after tumor cell inoculation based on the total flux (photons/second, minimum flux>1E6) and was performed on the basis of “Matched distribution” method (Study Director TM software, version 3.1.399.19). Treatment with vehicle (1% methylcellulose), IOA-244 (30 mg/mL, twice a day, orally) was initiated after grouping and continued throughout the experiment. Tumors were measured by bioluminescent imaging twice a week. At 15 minutes prior to imaging, d-Luciferin [PerkinElmer, XenoLight d-Luciferin (K+ salt), catalog no 122799] was injected into the animal at 150 mg/kg, i.p. and the mice were anaesthetized using isoflurane. Once the anesthesia had taken full effect, the bioluminescence was imaged using a living image program (PerkinElmer, IVIS Lumina Series III). tumor growth inhibition (TGI) was calculated, where TGI% is an indication of antitumor activity, and expressed as: TGI% = (1 − ΔT/ΔC)*100; ΔT/ΔC = (Ti − T0)/(Ci − C0) × 100% (Ti and Ci as the mean total flux (photons/second) of the treatment and vehicle groups on the measurement day; T0 and C0 as the mean total flux (photons/second) of the treatment and vehicle groups on the grouping day).

### Pharmacokinetic Studies

Pharmacokinetic parameters were determined in mouse and rat after single dosing (intravenous/oral administration) and were obtained from blood/feces/brain/cerebrospinal fluid samples collected from *in vivo* pharmacokinetic studies following standard Good Laboratory Practice (GLP) guidelines.

### GLP Toxicology Study in Rat

Wistar rats were administered with either vehicle [0.25% aqueous hydroxypopyl methylcellulose (Methocel K4M Premium)] or 30 mg/kg daily of the free base of IOA-244 (in the vehicle) via oral gavage for 28 days. Each group consisted of 5 male and 5 female rats. Additional satellite rats were dosed concurrently for the toxicokinetic evaluation of IOA-244.

Where appropriate, appearance, general condition, and behavior, in addition to mortality, were assessed daily. The bodyweight and food consumption of the animals were assessed at least once per week. Blood was collected from the animals and the following indexes were examined: red blood cells, platelets, immune cells, hemoglobin and mean cell volume, bilirubin, alanine aminotransferase, aspartate aminotransferase, alkaline phosphatase, and glutamate dehydrogenase (GLDH). All animals were anesthetized by carbon dioxide/air mixture and terminated via abdominal exsanguination on the scheduled last day of study.

In the event of an animal's death, from any cause, a necropsy was performed to assess gross pathologic alterations, organ weight, and histopathology. The study was conducted in accordance with GLP.

### Data Availability

Data are available at the NCBI GEO (http://www.ncbi.nlm.nih.gov/geo) database. *PIK3CD* expression values were also extracted from the data (GSE94669) previously obtained using a targeted RNA-seq approach, the HTG EdgeSeq Oncology Biomarker panel (HTG Molecular Diagnostics, Inc.; ref. 26). All the rest of data are available upon request to the authors.

## Results

### IOA-244 is a Selective and Metabolically Stable PI3Kδ Inhibitor

**FIGURE 1:**
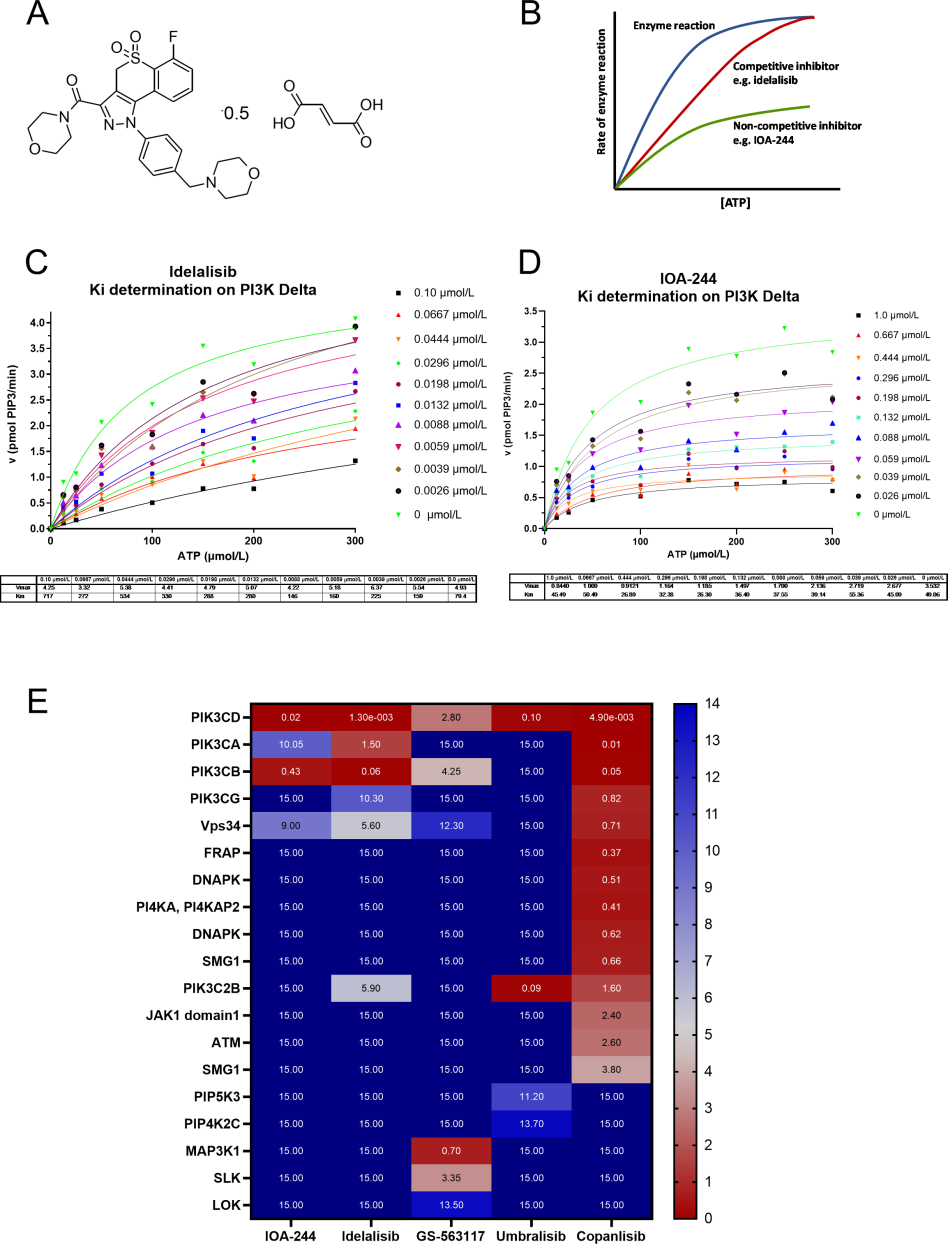
IOA-244 is a unique non–ATP-competitive, selective PI3Kδ inhibitor. **A,** Structural formula of IOA-244. **B,** Schematic Michaelis–Menten representation of an ATP-competitive versus a non–ATP-competitive inhibitor. Representative competition studies with idelalisib (**C**) and IOA-244 (**D**) on PI3Kδ varying in parallel compound and ATP concentrations. **E,** Heatmap presentation of the selectivity of selected PI3Kδ inhibitors and idelalisib main metabolite GS-563117 based on activity in Jurkat cell lysate as measured using the KiNativ method.

IOA-244 is the hemifumarate salt of MSC2360844, a compound with previously characterized pharmacokinetic and pharmacodynamic features (ref. 21; Fig. 1A; Supplementary Table S1). The compound inhibits PI3Kδ *in vitro* with an average IC_50_ value of 142 nmol/L, in line with its Ki value experimentally determined at 82 nmol/L. Competition studies indicated that IOA-244, differently from idelalisib, is a non–ATP-competitive inhibitor, a feature that has never been described for any PI3Kδ inhibitor (Fig. 1B–D).

For idelalisib, the first PI3Kδ inhibitor that entered clinical development (6, 7), the Km value for ATP in the SPA increased over 5-fold (from 64.4 to 347 μmol/L) with increasing compound concentration while the *V*_max_ value decreased less than 2-fold (from 4.53 to 2.48 pmol PIP3/minute). This is in line with the competitive inhibition toward ATP reported for this compound. On the other hand, for IOA-244, the Km value for ATP in the SPA assay increased less than 1.3-fold (from 37.6 to 45.5 μmol/L) with increasing compound concentration while the *V*_max_ value decreased almost 4-fold (from 3.14 to 0.84 pmol PIP3/minute), in line with a non-competitive mode of inhibition toward ATP (Fig. 1B–D). In addition, the IC_50_ values derived from this experiment, showed that, while for idelalisib the IC_50_ increased with increasing dose of ATP, the IC_50_ for IOA-244 remained constant (Supplementary Fig. S1A and S1B).

We then compared the on-target potency and selectivity of IOA-244 with idelalisib, umbralisib, and copanlisib, two other compounds with PI3Kδ inhibitory capacity, used in the clinic (6, 7). We profiled them in Jurkat cell lysate against a broad panel of protein and lipid kinases using the KiNativ platform (Fig. 1E; Supplementary Fig. S1C). While IOA-244 and umbralisib were highly stable in human microsomes (Supplementary Table S2), idelalisib is considerably metabolized in humans (27, 28). Therefore, GS-563117, the major metabolite of idelalisib with known inhibitory activity on LOK and SLK kinases (27, 28) was also analyzed. IOA-244 was confirmed to be selective for PI3Kδ with an average IC_50_ of 19 nmol/L. The only other kinases inhibited with an IC_50_ below 15 μmol/L were PI3Kβ (0.43 μmol/L), Vps34 (9.0 μmol/L), and PI3Kα (10.1 μmol/L). Similarly to IOA-244, in this assay also idelalisib was selective for PI3Kδ (1.3 nmol/L) over PI3Kβ (60 nmol/L), PI3Kα (1.5 μmol/L), Vps34 (5.6 μmol/L), PIK3C2B (5.9 μmol/L), and PI3Kγ (10.3 μmol/L). On the other hand, GS-563117 was not selective for PI3Kδ and showed the highest potency on MAP3K1 (0.7 μmol/L), followed by PI3Kδ (2.8 μmol/L), SLK (3.35 μmol/L), PI3Kβ (3,7 μmol/L), Vps34(12.3 μmol/L), and LOK (13.5 μmol/L). The selectivity profile of umbralisib was very different to those of IOA-244 and idelalisib with similar potency on PIK3C2B (90 nmol/L) and PI3Kδ (100 nmol/L) while further inhibiting PIP5K3 (11.2 μmol/L) and PIP4K2C (13.7 μmol/L; Fig. 1E). The reported potency of umbralisib on CK1ε was not confirmed in this assay (IC_50_ > 15 μmol/L; ref. 29). Compared with pan-PI3K inhibitor copanlisib, all three PI3Kδ inhibitors are more selective with respect to other lipid and protein kinases.

The same KiNativ platform was also used to measure the potency and selectivity of IOA-244 in human whole blood. IOA-244 was highly selective for PI3Kδ with an IC_50_ of 0.28 μmol/L. No other kinases were inhibited with an IC_50_ <30 μmol/L.

To confirm the biochemical isoform selectivity of IOA-244, idelalisib and umbralisib, the compounds were tested for growth inhibition of cancer cell lines especially sensitive to inhibition of specific PI3K class 1 isoforms: T-47D for PI3Kα, LNCAP for PI3Kβ, THP-1 for PI3Kγ, and SU-DHL-6 for PI3Kδ (Table 2; refs. 30–33). IOA-244 effectively inhibited proliferation of SU-DHL-6 with a GI_50_ of 0.24 μmol/L, whereas the only other cell line inhibited below 30 μmol/L was LNCAP (15 μmol/L). Idelalisib was highly potent on SU-DHL-6 (10 nmol/L) but also inhibited THP-1 (1.0 μmol/L), LNCAP (1.4 μmol/L), and T-47D (14 μmol/L). Umbralisib was the least potent inhibitor on SU-DHL-6 (1.5 μmol/L), and inhibited THP-1 (11 μmol/L), LNCAP (11 μmol/L) and T-47D (26 μmol/L; Table 2). Overall, these data indicate that IOA-244 is a unique PI3Kδ inhibitor with good biochemical and cellular selectivity (Supplementary Fig. S2).

**TABLE 2:** Comparison of PI3K isoform specificity for IOA-244 and other PI3Kδ inhibitors

	Parameter	IOA-244	Idelalisib	GS563117	Umbralisib
PI3K isoform selectivity	IC_50_ PI3Kα	10.1 μmol/L	1.5 μmol/L	>15 μmol/L	>15 μmol/L
	IC_50_ PI3Kβ	0.43 μmol/L	51 nmol/L	3.7 μmol/L	>15 μmol/L
	IC_50_ PI3Kγ	>15 μmol/L	10.3 μmol/L	>15 μmol/L	>15 μmol/L
	IC_50_ PI3Kδ	19 nmol/L	1.3 nmol/L	2.7 μmol/L	0.1 μmol/L
Cellular sensitivity	GI_50_ T-47D (PI3Kα)	>30 μmol/L	14.1 μmol/L	NA	26.3 μmol/L
	GI_50_ LNCaP (PI3Kβ)	15 μmol/L	1.35 μmol/L	NA	11.2 μmol/L
	GI_50_ THP-1 (PI3Kγ)	>30 μmol/L	1.03 μmol/L	NA	11.4 μmol/L
	GI_50_ SU-DHL-6 (PI3Kδ)	0.235 μmol/L	10 nmol/L	NA	1.51 μmol/L

NOTE: IC_50_ determined using the Kinativ assay in Jurkat cell lysate, upon treatment with increasing doses of the indicated molecules. GI_50_ was determined in the indicated cell lines upon treatment with increasing doses of the indicated molecules.

### *In Vivo* Pharmacokinetics and Pharmacokinetic/pharmacodynamic Relationship

Pharmacokinetic parameters of IOA-244 were determined in mouse and rat, after single dosing of the compound (Table 3). Mouse oral bioavailability was good, with 79% in males and 66% in females. In rat, the oral bioavailability including that following capsule dosing was very good at 109% in males and 71%–111% in females. Compared with mouse, the exposure in rat was 1.2- and 1.9-fold higher with respect to total and free *C*_max_ and 3- and 5.6-fold higher with respect to total and free AUC. The recovery of compound in the feces was low, suggesting that the compound had a good absorption, and it was not a P-gp substrate. A biodistribution study in mouse showed that IOA-244 was well distributed in all tissues investigated, including brain.

**TABLE 3:** Pharmacokinetic parameters for IOA-244 in mouse and rat

Species	Mouse		Rat	
Sex	Male	Female	Male	Female
Dose (mg/kg)	1		1	
CLP (L/hour/kg)	1.43	1.43	0.502	0.351
*V*ss (L/kg)	1.92	1.71	2.45	2.19
*t*1/2 (hours)	1.24	1.42	4.52	5.13
Dose (mg/kg)	5		5	
*F* (%)	79	66	109	102
Dose (mg/kg)	30		30^a^	
Total *C*_max_ (ng/mL)	6,647	—	8,045	8,965
Free *C*_max_ (ng/mL)^b^	3,390	—	6,114	6,813
Total AUC (hour × ng/mL)	13,480	—	44,450	57,150
Free AUC (hour × ng/mL)^b^	6,875		33,782	43,434

^a^Toxicokinetics data at day 28.

^b^Estimated on the basis of protein binding of IOA-244 in mouse and rat plasma of 51% and 76%, respectively as measured by ultrafiltration.

The relationship between dosing regimen, exposure, and pharmacodynamic effects (pAkt) of IOA-244, as well as the construction of a mathematical pharmacokinetic/pharmacodynamic model has been reported before (21). On the basis of this model, 50% pAkt inhibition over 24 hours is sufficient to achieve maximal disease modulation. With a reported pAkt IC_50_ of 463 nmol/L in mouse whole blood, twice daily dosing of IOA-244 in mouse at 30/mg/kg/day is predicted to overcome this threshold.

### IOA-244 Limits Proliferation of Lymphoma Cancer Cells Expressing High Levels of PI3Kδ

**FIGURE 2:**
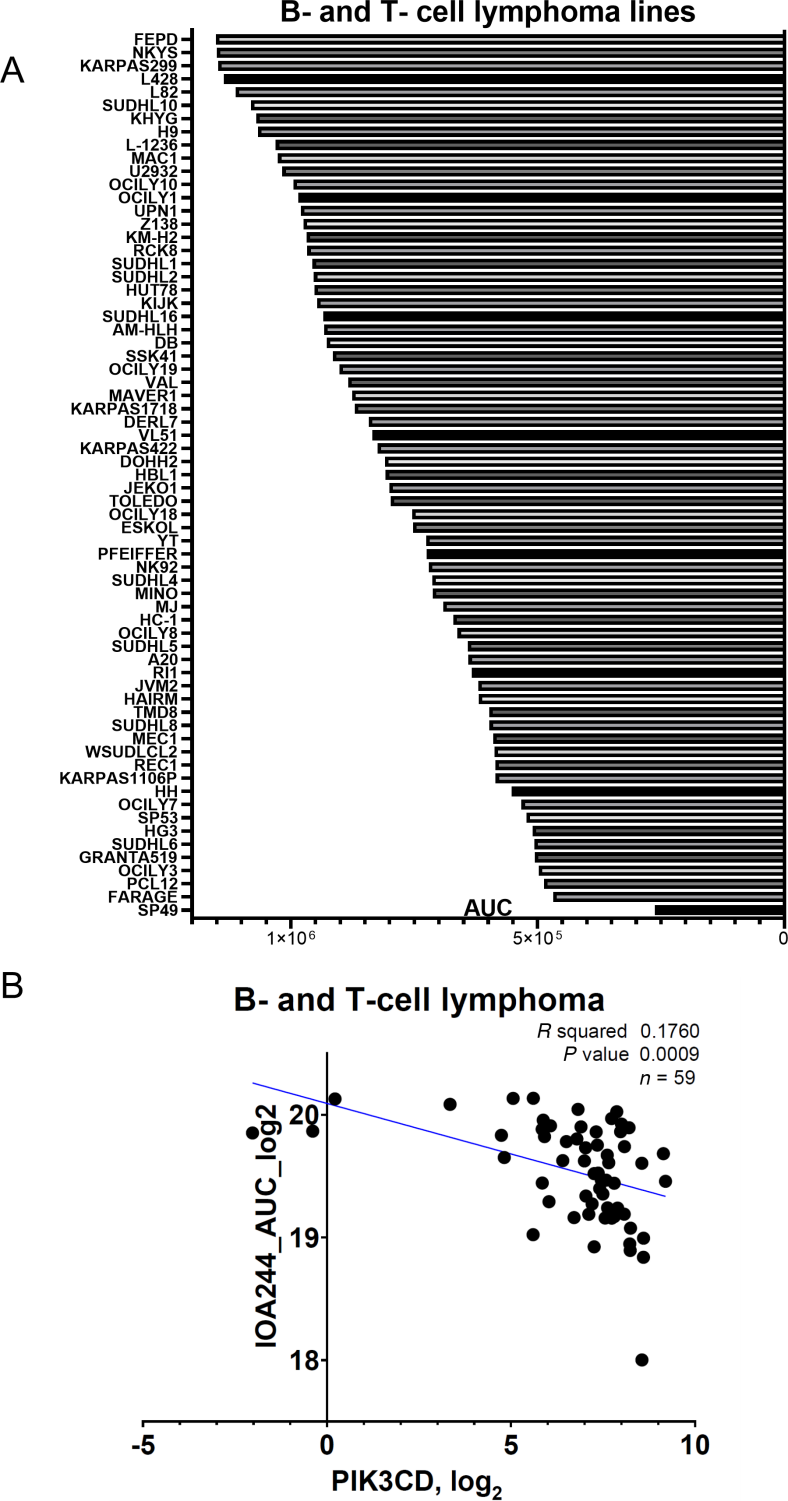
Tumor-intrinsic effect of IOA-244. **A,** Activity of IOA-244 in 67 human lymphoma cell lines. AUC was calculated after IOA-244 treatment at increasing concentration (13.7 nmol/L to 10 μmol/L; 1- to 3-fold dilution) of the drug for 72 hours with Prism software v8.0 (GraphPad). **B,** Correlation between AUC and *PIK3CD* transcript expression in 59 B and T-cell lymphoma cell lines. AUC was calculated after IOA-244 treatment at increasing concentration of the drug for 72 hours; *PIK3CD* RNA expression was extrapolated by RNA-seq of each cell line at baseline conditions. *R*^2^, Spearman correlation.

As PI3Kδ inhibitors have been originally exploited in the context of hematologic cancers, we tested the efficacy of IOA-244 in a large collection of 67 B- and T-cell lymphoma cell lines, representing different pathologic subsets (Supplementary Table S3). Cell lines were exposed to increasing concentrations of IOA-244 (from 13.7 nmol/L to 10 μmol/L) and proliferation was assessed after 72 hours by MTT assay. For each cell line, a dose–response proliferation curve was generated, and the AUC calculated (Fig. 2A). These data showed that there was heterogeneity in the response to IOA-244 across cell lines and we wondered whether the observed effect could be at least partially explained by different expression levels of PI3Kδ. Thus, we correlated the AUC with the mRNA expression levels of *PIK3CD* measured via total RNA-seq. This analysis demonstrated that the antitumor activity of the molecule positively and significantly correlated with the expression levels of PI3Kδ (*R*^2^ = 0.18, *P* = 0.0009; Fig. 2B). Considering the possible application in the clinical context, we also analyzed the correlation with PI3Kδ expression values in a subset of 41 B- and T-cell lymphoma cell lines, previously profiled (GSE94669) with a targeted RNA-seq approach (HTG EdgeSeq Oncology Biomarker panel) specifically designed for the analysis of formalin-fixed paraffin-embedded tissues. Also in this case, IOA-244 antitumor activity and *PIK3CD* expression values were significantly correlated (*R*^2^ = 0.19, *P* = 0.005; Supplementary Fig. S3A).

To expand on the differentiation of IOA-244 from the ATP-competitive inhibitors, we took advantage of the antitumor activity data previously obtained for idelalisib (26). Considering the B- and T-cell lymphoma cell lines exposed to both PI3K inhibitors (*n* = 41), the correlation between AUC values and *PIK3CD* mRNA expression was stronger for IOA-244 (*R*^2^ = 0.19, *P* = 0.005), than for idelalisib (*R*^2^ = 0.11, *P* = 0.033; Supplementary Fig. S3A and S3B). Focusing on the B-cell lymphoma cell lines (*n* = 36), IOA-244 still correlated with PI3Kδ levels (*R*^2^ = 0.14, *P* = 0.02), but not idelalisib (*R*^2^ = 0.05, *P* = 0.17; Supplementary Fig. S3C and S3D).

Overall, our data demonstrate that IOA-244 has efficacy and on-target intrinsic activity in B-cell lymphomas.

### Immunomodulatory Properties of IOA-244

**FIGURE 3:**
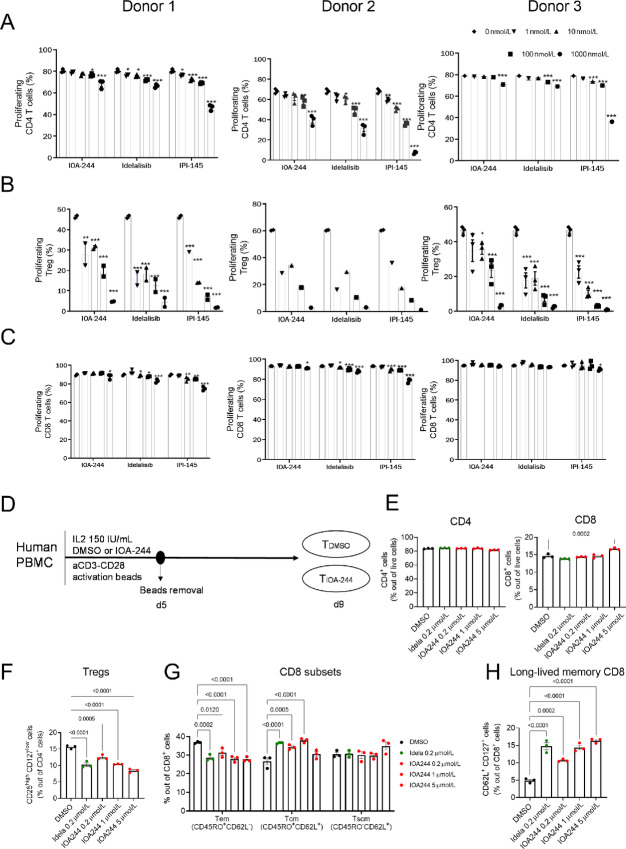
Dose-dependent inhibition of T_reg_ proliferation by IOA-244. Proliferation of CD4^+^ Tconv cells (**A**), Tregs (**B**), and CD8^+^ T cells (**C**) proliferation was analyzed by flow cytometry 5 days after stimulation with αCD3/αCD28 or resting, alongside IL2 and compounds at a range of concentrations and IL2. Dilution of ef450 proliferation dye was used to determine the frequency of cells that had undergone cellular division. Three donors were used (*n* = 3) and the statistics shown are two-way ANOVAs relative to the 0 nmol/L control group (*, *P* < 0.05; **, *P* < 0.01; ***, *P* < 0.001). **D,** Experimental layout. **E,** Percentage of CD4 and CD8 T cells out of live cells (*n* = 3 human donors/group). **F,** Percentage of Tregs out of CD4 population (measured by CD25high-CD127Low). **G,** Percentage of CD8T effector memory (Tem), CD8T central memory (Tcm), and CD8T stem-cell memory (Tscm) as measured by gating with CD45RO and CD62L. **H,** Percentage of long-lived memory CD8^+^ T cells, measured by coexpression of CD127 and CD62L. Statistics shown are one-way or two-way ANOVAs (**G**) relative to the DMSO vehicle group.

The effect of IOA-244 on B-cell signaling pathways, T-cell cytokines, and primary cell cocultures has been described previously (21). To test the selective inhibition of T_reg_ proliferation by PI3Kδ inhibitors, human CD4^+^CD25^+^ (T_reg_), CD4^+^ and CD8^+^ T cells were isolated from healthy volunteer blood donors. We tested the ability of the PI3Kδ isoform inhibitor compounds, IOA-244, idelalisib and the dual PI3Kγ/δ inhibitor duvelisib (IPI-145) to modulate the proliferation of each population of T cells in response to anti-CD3/anti-CD28 stimulation *in vitro* (Fig. 3A–C). Stimulated T_reg_ proliferative responses were significantly reduced in the presence of all three inhibitors at all doses and in all donors (Fig. 3B). This effect was dose dependent (IC_50_ 100–300 nmol/L) and maintained even at the lowest concentration of inhibitor (1 nmol/L). Stimulated conventional CD4^+^ T-cell proliferative responses were only marginally reduced in the presence of IOA-244 at concentrations ranging from 1 to 100 nmol/L (Fig. 3A). Furthermore, at the high concentration (1 μmol/L), proliferative responses were only slightly reduced whereas in contrast, high-dose IPI-145 greatly reduced conventional T-cell proliferative responses (Fig. 3A). No inhibition of CD8^+^ T-cell proliferation was seen in the presence of IOA-244, whereas there was a small but measurable effect of idelalisib and a slightly greater effect of duvelisib, likely to be attributable to effects on PI3Kγ (Fig. 3C). These data strongly support the hypothesis that CD8^+^ T cells were only marginally sensitive to PI3Kδ inhibition by IOA-244, while T_reg_ proliferative responses were significantly reduced (Fig. 3A–C). Recent discoveries have highlighted the importance of PI3Kδ in regulating CD8 T-cell activation, by promoting effector functions, while inhibiting the differentiation toward a memory-like phenotype (34–36). Mechanistically, PI3K-activated Akt phosphorylates FOXO and sequester it in the cytosol, therefore inhibiting the transcription of CD62L, CD127, and CCR7, molecules involved in the generation of central memory and stem-like memory T cells (37). Interestingly, memory differentiation of CD8 T cells can prevent T-cell exhaustion, ensuring greater expansion capacity and a long lasting antitumoral immune response (2). In addition to that, differently from exhausted CD8 T cells, progenitor stem-cell like CD8 T cells in solid tumors respond to checkpoint inhibitor therapies (38, 39). To explore the effect of IOA-244 on T-cell activation and differentiation, peripheral blood T cells from healthy donors were activated in presence of increasing doses of IOA-244 and analyzed at day 9 by flow cytometry (Fig. 3D). Idelalisib was used as positive control, as it was previously shown to promote memory-like differentiation of *ex vivo* treated T cells and increase their antitumoral function during adoptive cell transfer (34, 35). Confirming our previous observation, IOA-244 did not affect CD4 and CD8 T-cell expansion (Fig. 3E), but it reduced the percentage of T_reg_ within the CD4^+^ T cells in dose-dependent manner and similarly to idelalisib (Fig. 3F). In addition to that, IOA-244 decreased effector memory CD8 T cells, while increasing central memory at lower doses and stem cell memory CD8 T cells at the highest dose tested (Fig. 3G). Also, there was a significant effect in augmenting the CD62L/CD127^+^ CD8 T cells, described as long-lived CD8 T cells (Fig. 3H). These data highlight the promising therapeutic potential of IOA-244 treatment in cancer types in which a high burden of T_regs_ and exhausted CD8 T cells make these tumors resistant to immunotherapies.

### IOA-244 Remodels the Tumor Microenvironment and Potentiates Checkpoint Inhibitor Efficacy

**FIGURE 4:**
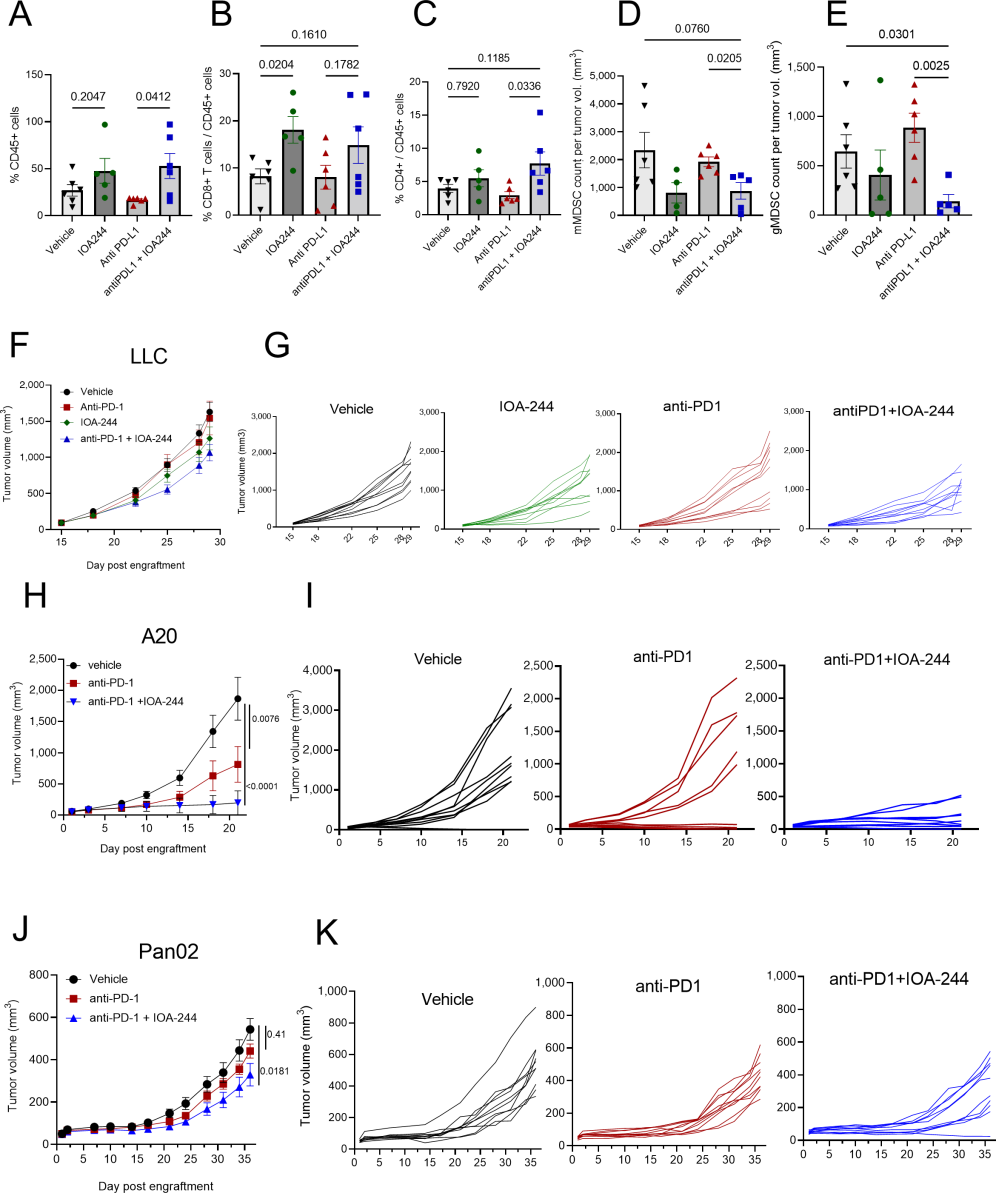
IOA-244 remodels the immune microenvironment and allow ICB-driven antitumor immune response. Flow cytometry analysis from CT26 tumors showing quantification of CD45^+^ cells (**A**), CD8/CD45 (**B**), CD4/CD45 (**C**), mMDSC/CD45 (**D**), gMDSC/CD45 (**E**). **F** and **G,** Efficacy of IOA-244 alone or combined with anti-PD-L1 in the LLC (**H** and **I**), A20 (**H–I**) and Pan-02 (**J** and **K**) syngeneic mouse tumor models, measuring tumor volumes. Statistics shown are two-way ANOVAs (Tukey multiple comparison test).

To test the efficacy of IOA-244 i*n vivo*, we selected four different syngeneic mouse cancer models. On the basis of the *in vitro* results, showing immunomodulatory effects of IOA-244, we reasoned that, in addition to monotherapy, combination with immunotherapy might result in increased therapeutic benefit in tumors normally unresponsive to immune checkpoint blockade (ICB). The subcutaneous colorectal cancer CT26 model was used to test the efficacy of IOA-244 alone (30 mg/kg twice daily, orally) and in combination with anti-PD-L1 (10 mg/kg once every other day, i.p.), which was also included as a monotherapy control arm (Supplementary Fig. S4A). While there was no improvement upon monotherapy with both agents, the combination of IOA-244 with anti-PD-L1 demonstrated a reduction, albeit not significant, in tumor growth over time (Supplementary Fig. S4A). Following 20 days of treatment, the combination of IOA-244 with anti-PD-L1 significantly enhanced the proportion of CD45^+^ cell infiltrates when compared with anti-PD-L1 treatment alone (Fig. 4A). Combination treatment also significantly enhanced the proportion of infiltrating CD8^+^ T cells compared with vehicle control and anti-PD-L1 monotherapy groups (Fig. 4B). Moreover, a significant increase in the proportion of CD4^+^ T-cell infiltrates was observed in the combination treatment group when compared with IOA-244 alone and anti-PD-L1 alone (Fig. 4C). Compared with the anti-PD-L1-only treatment group, the number of monocytic (mMDSC) and granulocytic (gMDSC) MDSCs was significantly reduced following combination treatment (Fig. 4D and E). Finally, compared with anti-PD-L1 treatment alone, the combination of anti-PD-L1 plus IOA-244 significantly enhanced the frequency of natural killer (NK) cell infiltrates (Supplementary Fig. S4B).

Taken together, the data from the CT26 model support the antitumor efficacy of IOA-244 in combination with ICB and demonstrate that IOA-244 alone or in combination with immunotherapy can modulate the composition of tumor-infiltrating immune cells toward an antitumor phenotype.

The syngeneic subcutaneous LLC mouse model was used to compare the efficacy of IOA-244 (30 mg/kg, twice a day, orally) alone or in combination with anti-PD-1 (10 mg/kg, twice a week, i.p.). Anti-PD-1 was also tested as a monotherapy. A proportion (4/10) of animals showed a moderate response to anti-PD-1 therapy (Fig. 4F and G), corresponding to a 9% reduction in the area under the concentration-versus-time curve (AUC) of tumor growth over the time course of the study (Supplementary Fig. S4C). Treatment with IOA-244 alone resulted in a trend of delayed tumor growth, with a 24% reduction in the AUC of tumor growth. The combination of anti-PD-1 and IOA-244 had the strongest effect on tumor growth with a slower rate of growth and a reduction of 36% of the AUC (Fig. 4F and G; Supplementary Fig. S4C). None of the effects reached statistical significance, but they do suggest that IOA-244 alone and in combination with anti-PD-1 influenced the tumor growth rate in this model. No analysis of tumor-infiltrating lymphocytes was performed in this model. However, historical data suggest that this model is dependent on MDSCs to inhibit the antitumor immune response, suggesting that IOA-244 may be modulating the MDSC population to support stronger antitumor immunity (40, 41).

The A20 lymphoma model was first used to assess IOA-244 treatment as a monotherapy. While there was not a significant reduction of the tumor burden, IOA-244 delayed the tumor growth and progression (Supplementary Fig. S4D and S4E). We then tested the combination efficacy of IOA-244 and anti-PD-1, all animals treated with the combination showed strong inhibition of tumor growth compared with animals (5/10) in the anti-PD-1 alone treated group (Fig. 4H and I). These data suggest that IOA-244 was able to increase the efficacy of checkpoint blockade inhibitors, by promoting a functional antitumor immune response.

Similar data were obtained in the pancreatic Pan-02 syngeneic model in which the combination of IOA-244 with anti-PD-1 showed a greater effect on the reduction of tumor growth compared with anti-PD-1 alone with 4 of 10 animals showing an improvement in the response and 1 of 10 showing a complete tumor regression (Fig. 4J and K). Monotherapy of IOA-244 in an orthotopic pancreatic model derived from the genetically engineered KPC mouse model showed only modest effects on tumor growth (Supplementary Fig. S4F and S4G). Overall, these preclinical data indicate that IOA-244 is a safe and specific PI3Kδ inhibitor with immunomodulatory features and cytotoxic activity in tumors expressing high levels of the target.

### IOA-244 is Well Tolerated in Rat at Supratherapeutic Exposure Levels

The toxicokinetic analysis showed that the IOA-244 exposure levels in the toxicology study at the 30 mg/kg dose level were more than the exposures measured in mouse at the corresponding dose level also when considering twice a day dosing (Table 3). Furthermore, no compound accumulation was observed between day 1 and day 28 of the study.

There were no clinical observations in the vehicle or IOA-244–treated animals. There were several changes in circulation in the IOA-244–treated animals that were all considered minimal: leucocytes were slightly decreased, mainly due to a drop in lymphocytes (slight decrease of absolute and relative lymphocyte number, respectively B lymphocytes); the relative numbers of neutrophilic granulocytes were slightly increased. Although all hematologic alterations were within the internal laboratory control range, they had to be considered as treatment related. Clinical chemistry evaluation revealed in female rats a slight increase of total bilirubin as well as increased GLDH. The changes were within internal laboratory ranges.

Gross pathology as well as body and organ weights of animals at the end of the study were inconspicuous. Histopathology findings in single IOA-244–treated rats were mainly restricted to the B-cell compartments of lymph nodes and spleen showing a decrease of germinal center formation and a depletion of the marginal zone. These were not considered to represent adverse findings but to reflect the pharmacologic properties of IOA-244. As other PI3Kδ inhibitors have been known to cause histopathologic changes in the testes, a special focus was set on investigation of this potential target organ. However, testes from IOA-244–treated animals, investigated with hematoxylin and eosin and periodic acid-Schiff staining, did not reveal any lesions.

## Discussion

Here, we presented and characterized the first-in-class non–ATP-competitive and selective PI3Kδ inhibitor IOA-244, showing its specificity for the PI3Kδ isoform and its direct and indirect (immune-mediated) antitumor activity in various tumor models.

PI3Kδ knockout mice have immune deficiencies but are fertile and healthy (2), suggesting that PI3Kδ is a valid pharmacologic target for immune-mediated diseases such as autoimmune, inflammatory diseases, and cancer (42–44). However, fully exploiting these opportunities has, so far, been restrained by the challenge of discovering inhibitors with an acceptable safety profile.

To our knowledge, IOA-244 is the first non–ATP-competitive PI3Kδ inhibitor. While the exact mechanism of its binding is under investigation, generally non–ATP-competitive kinase inhibitors induce a conformation shift in the enzyme by binding (at least partially) to an allosteric pocket (45). Allosteric inhibitors typically have the potential for higher selectivity, lower toxicity, and improved physicochemical properties compared with conventional kinase inhibitors. Although the exact biological consequences of non–ATP-competitive PI3Kδ inhibition are currently being evaluated, early clinical data (22, 23) suggest that IOA-244 is better tolerated in patients compared with ATP-competitive inhibitors.

Furthermore, IOA-244 is a selective inhibitor in both biochemical and physiologically relevant assays (21). The IC_50_ of IOA-244 in whole blood is 280 nmol/L. Taking into account the 10-fold dilution of cell pellets during lysis prior to the target engagement analysis, the true IC_50_ could be as low as 28 nmol/L, which is similar to the IC_50_ observed in the Jurkat cells lysate of 19 nmol/L. In contrast, the PI3Kδ inhibitor umbralisib was less selective than reported (29) and can be considered a dual PIK3C2B/PI3Kδ inhibitor in cellular assays. Idelalisib showed a selectivity profile similar to IOA-244. However, in patients idelalisib generates the pharmacologically active metabolite GS-563117, as well as reactive metabolites (27, 28, 46). Although this metabolite showed low potency in the Jurkat cell lysate, its high clinical exposure [average *C*_trough_ is approximately 5.8 μmol/L (47)] and broad activity profile might contribute to off-target toxicities of idelalisib. In contrary, IOA-244, similarly to umbralisib, is metabolically stable and the absence of reactive metabolites is expected to result in an improved safety profile. Indeed, the formation of reactive metabolites is another explanation for the reported side effects of idelalisib and copanlisib (47, 48).

The specificity and selectivity of IOA-244 is associated with the expected effects on its signaling pathway, such as pAKT in B cells, CD63 expression on basophils and modulation of B- and T-cell function (21). Here, we show that IOA-244 also decreased proliferation of lymphoma cell lines and the antitumor activity was dependent on PI3Kδ.  Indeed, the antiproliferative activity of the molecule positively correlated with the RNA expression levels of *PIK3CD*, measured with two different approaches. Of note, the activity of idelalisib in lymphoma cell lines was much less correlated with the expression levels of the target. These data suggest that IOA-244 holds great potential to be used in hematologic cancers similar to other PI3Kδ inhibitors. The ongoing and the future clinical trials will show whether the unique features and safety profile of IOA-244 will distinguish its clinical activity and mechanism of action from the other inhibitors.

PI3Kδ sustains specifically the activity and proliferation of immunosuppressive cells as T_reg_ and MDSCs (49). While the exact molecular mechanism is unknown, this harbors enormous therapeutic potential for targeting PI3Kδ in solid tumors where the harsh microenvironment particularly favors suppressive cells, inhibiting cytotoxic activity of CD8T cells and proinflammatory myeloid cells. In line with other PI3Kδ inhibitors, IOA-244 inhibited T_reg_ proliferation with an IC_50_ value comparable with other selective PI3Kδ inhibitor, while having limited antiproliferative effects on CD4^+^ T cells other than T_reg_ and no effect on CD8^+^ T cells. Moreover, IOA-244 increased the differentiation of activated CD8 T cells toward a memory-like phenotype. In cancer, a pool of progenitor memory-like CD8 T cells has been shown to prevent exhaustion, while preserving a great expansion capacity, cytotoxicity and long lasting antitumoral immunity (38). Whether *in vivo* treatment of IOA-244 will have similar effects on the CD8 T-cell subsets remain to be determined. According to the activity observed *in vitro*, IOA-244 treatment in immune-competent tumor-bearing mice, was able to remodel the tumor microenvironment. In the CT26 colon cancer model, IOA-244 administration decreased MDSCs while it increased CD8^+^ T cells and NK cells in the tumor microenvironment, hereby enhancing the efficacy of anti-PD-L1 treatment. In the LLC syngeneic mouse lung cancer model, IOA-244 inhibited tumor growth in monotherapy and sensitized the tumors to anti-PD-1 treatment. A sensitization to PD-1 treatment was also observed in the pancreatic cancer (Pan-02) and B-cell lymphoma (A20) syngeneic mouse models. The lower activity of IOA-244 as single agent in this model compared with parsaclisib (50), another highly selective PI3Kδ inhibitor, is probably explained by the suboptimal dosing schedule used with dosing holidays over the weekend. Overall, these findings support the use of IOA-244 in combination with checkpoint inhibitors in solid tumors. In addition to that, selections of tumor indications harboring high expression levels of PI3Kδ might unleash a double mode of action, where intrinsic antitumoral activity will combine with extrinsic effects on immune cells to boost antitumoral response (9, 11, 51).

The main target organ for toxicity in rats exposed to 30 mg/kg/day of IOA-244 for 28 consecutive days was restricted to the B-cell compartment in lymph nodes and spleen. These effects are likely on-target and reflect the inhibition of PI3Kδ by IOA-244. These findings were incorporated into the design of the phase I/II clinical evaluation of IOA-244.

In conclusion, we presented the preclinical immunology, oncology, and toxicology data on the first-in-class, selective non–ATP-competitive PI3Kδ inhibitor IOA-244, which supported the design of the ongoing phase I study (NCT04328844) for patients with hematologic or solid tumors expressing high levels of PI3Kδ.

## Supplementary Material

Figure S1 IC50 curve of the ATP competitive assay and pie chart of the Kinativ assay

Figure S2 Target engagement kinase tree

Figure S3 Correlation analysis of PI3Kd levels and the response to IOA-244 and Idelalisib

Figure S4 Tumor experiments showing monotherapy testing of IOA-244

Table S1 Table summarizing the IC50 values derived from previous published experiments performed with IOA-244

Table S2 Table showing values of microsomal stability of IOA-244

Table S3 Table showing the raw data of the AUC value of figure 2A
