# Supplementary material for: IOA-244 is a Non–ATP-competitive, Highly Selective, Tolerable PI3K Delta Inhibitor That Targets Solid Tumors and Breaks Immune Tolerance
Source: Cancer Res Commun. 2023 Apr 14;3(4):576–91. doi: 10.1158/2767-9764.CRC-22-0477 (PMC10103717; doi:10.1158/2767-9764.CRC-22-0477)
Supplement: Figure S1 — IC50 curve of the ATP competitive assay and pie chart of the Kinativ assay [file crc-22-0477-s01.pdf]

A

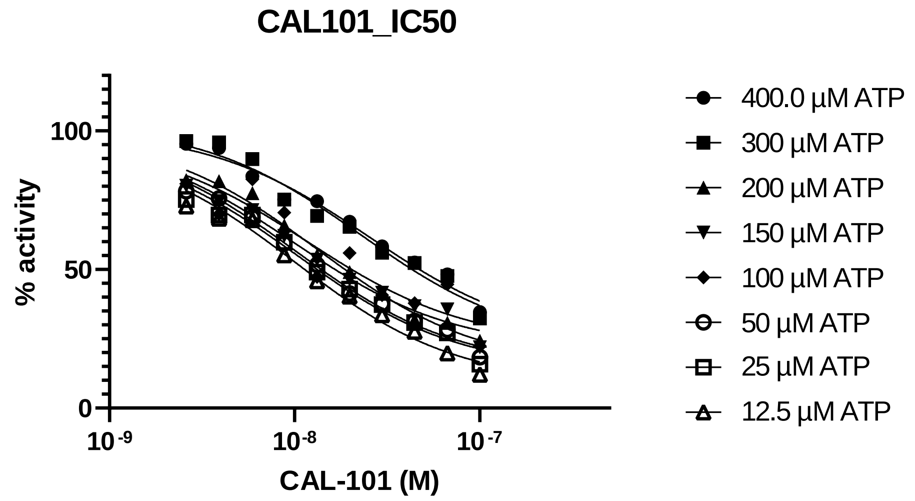

	400.0 µM ATP	300 µM ATP	200 µM ATP	150 µM ATP	100 µM ATP	50 µM ATP	25 µM ATP	12.5 µM ATP
IC50	2.515e-008	2.285e-008	1.343e-008	9.878e-009	1.163e-008	1.019e-008	1.031e-008	1.011e-008

B

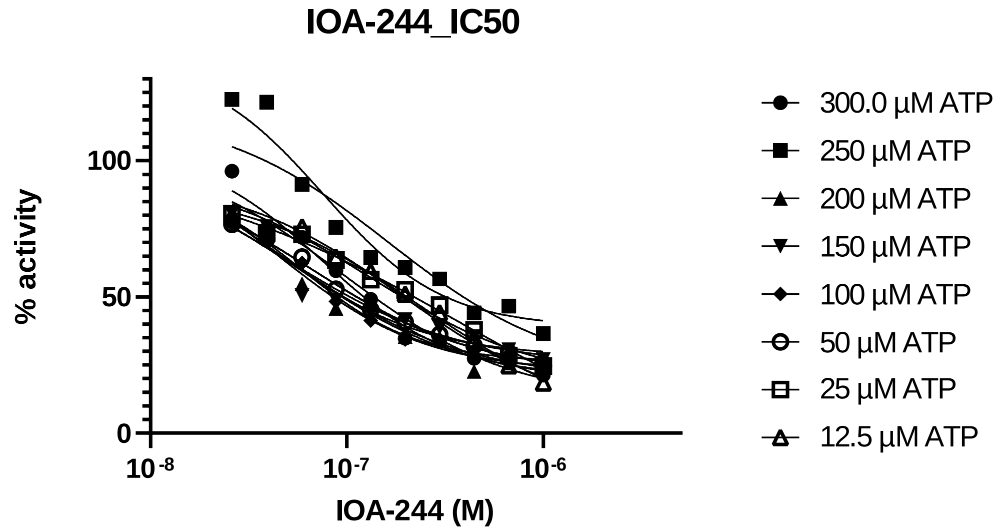

	300.0 µM ATP	250 µM ATP	200 µM ATP	150 µM ATP	100 µM ATP	50 µM ATP	25 µM ATP	12.5 µM ATP
IC50	9.449e-008	1.635e-007	5.782e-008	4.956e-008	5.649e-008	6.420e-008	1.310e-007	1.885e-007

C

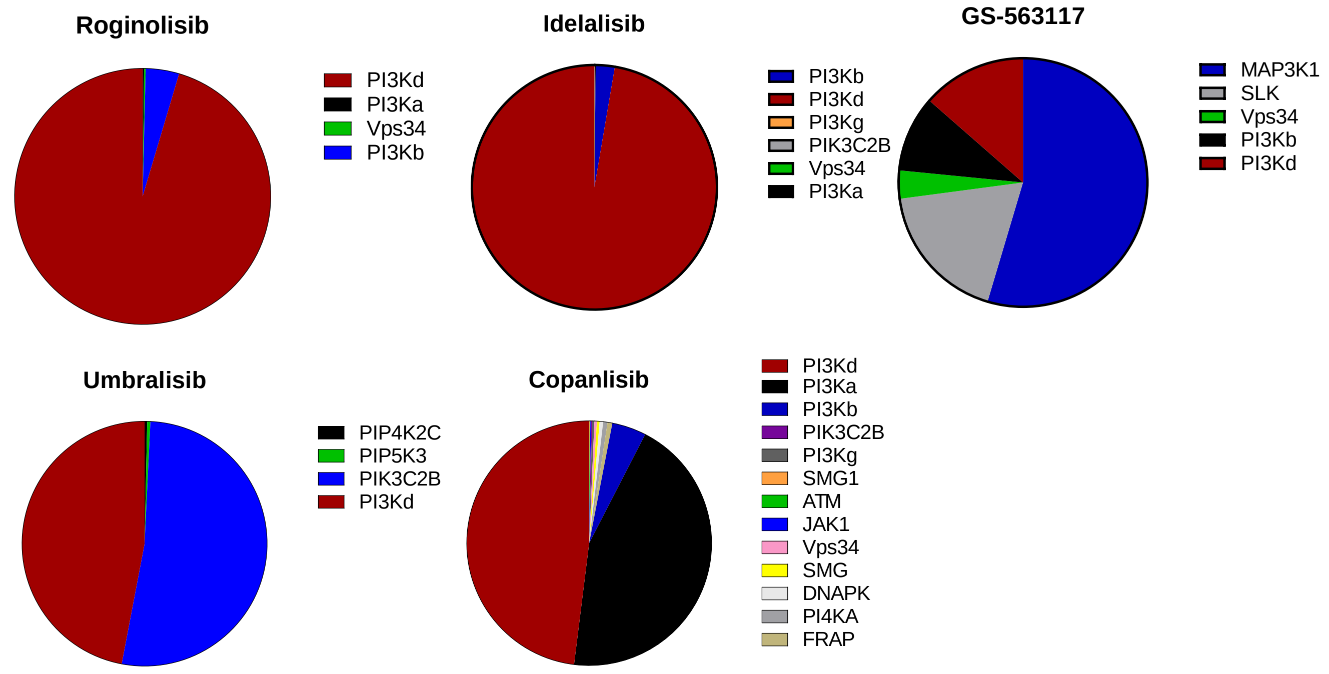

Supplementary figure 1: (A-B) IC50 of Idelalisib (A) and IOA-244 (B) derived from the ATP competition assay shown in Figure 1C and 1D respectively. (C) Representation of the selectivity of the represented PI3Kδ inhibitors and Idelalisib main metabolite GS-563117 using partition index based on IC50 values in Jurkat cell lysate as measured by KiNativ assay.
