# Supplementary figures and images for: IOA-244 is a Non–ATP-competitive, Highly Selective, Tolerable PI3K Delta Inhibitor That Targets Solid Tumors and Breaks Immune Tolerance
Source: Cancer Res Commun. 2023 Apr 14;3(4):576–91. doi: 10.1158/2767-9764.CRC-22-0477 (PMC10103717; doi:10.1158/2767-9764.CRC-22-0477)

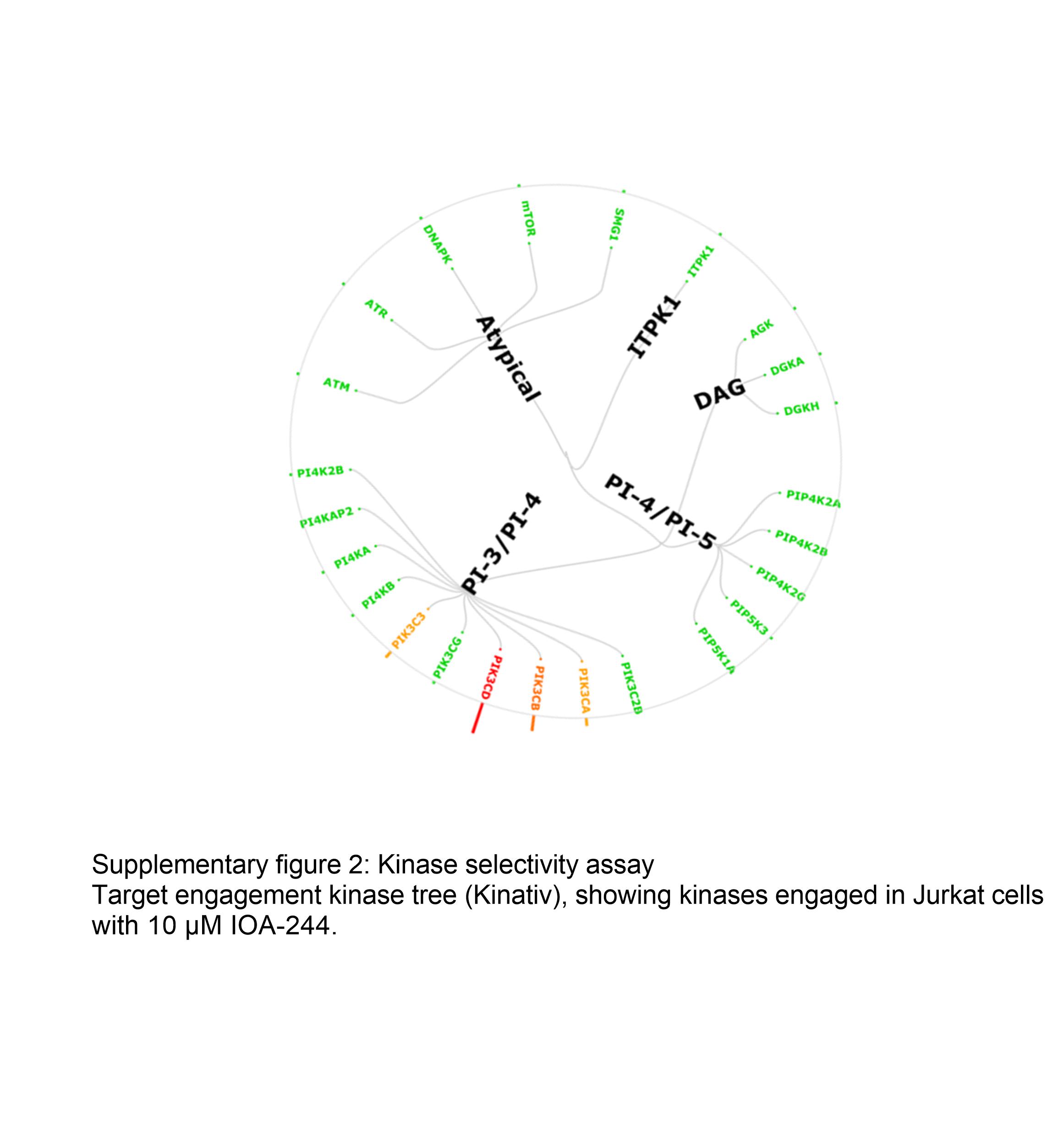

Supplement: Figure S2 — Target engagement kinase tree [file crc-22-0477-s02.jpeg]
