# Supplementary material for: IOA-244 is a Non–ATP-competitive, Highly Selective, Tolerable PI3K Delta Inhibitor That Targets Solid Tumors and Breaks Immune Tolerance
Source: Cancer Res Commun. 2023 Apr 14;3(4):576–91. doi: 10.1158/2767-9764.CRC-22-0477 (PMC10103717; doi:10.1158/2767-9764.CRC-22-0477)
Supplement: Figure S3 — Correlation analysis of PI3Kd levels and the response to IOA-244 and Idelalisib [file crc-22-0477-s03.pdf]

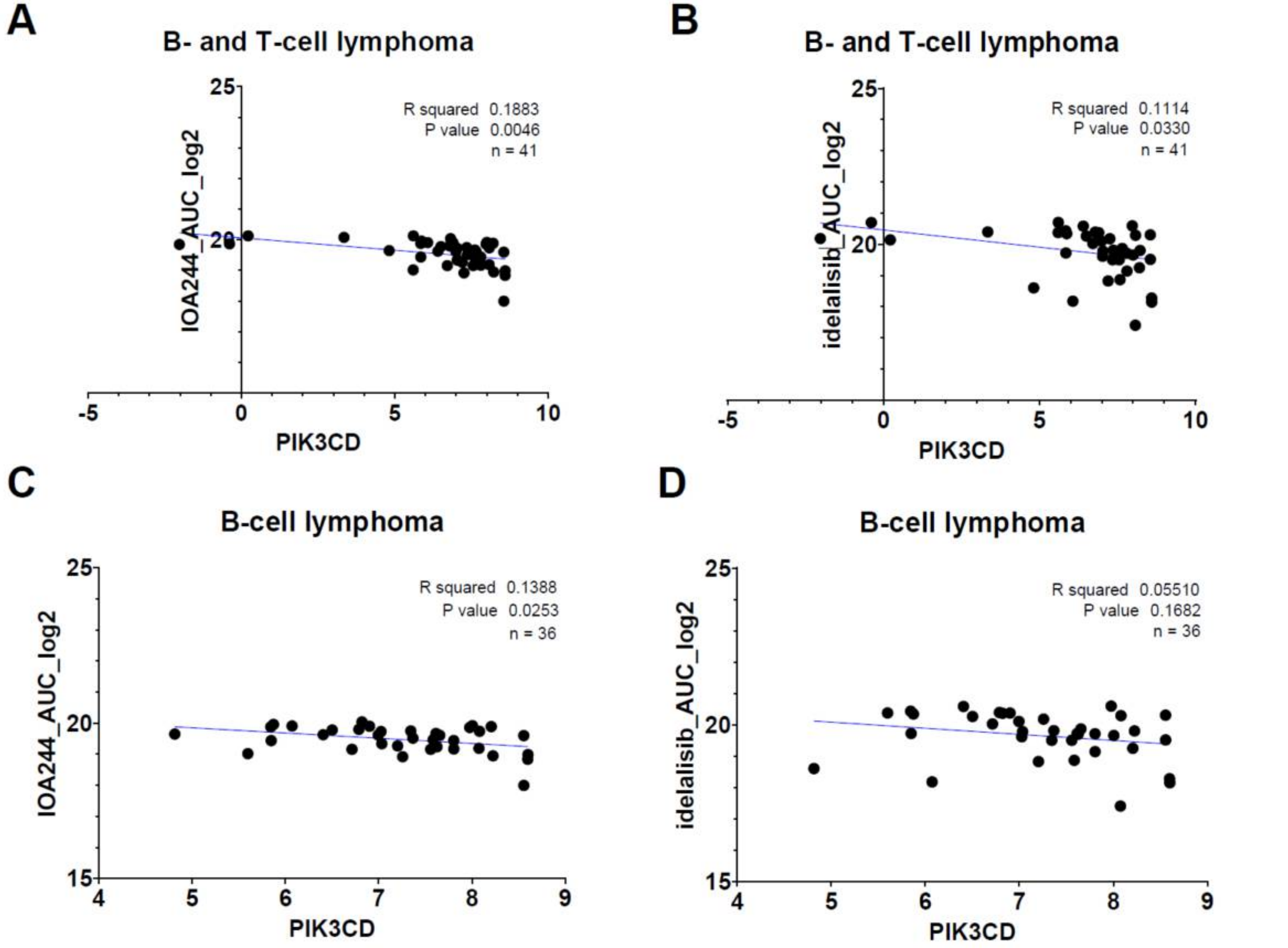

Supplementary figure 3**:** (A-D) Correlation between AUC values and PIK3CD transcript expression in 41 B and T cell lymphoma cell lines exposed to IOA-244 (A) or to idelalisib (B) and in 36 B cell lymphoma cell lines exposed to IOA-244 (C) or to idelalisib (D). AUC was calculated after treatment with the drugs at increasing concentrations for 72 hours; PIK3CD RNA expression was extrapolated by RNA sequencing of each cell line at baseline conditions. R^2, Spearman correlation.
