# Supplementary material for: IOA-244 is a Non–ATP-competitive, Highly Selective, Tolerable PI3K Delta Inhibitor That Targets Solid Tumors and Breaks Immune Tolerance
Source: Cancer Res Commun. 2023 Apr 14;3(4):576–91. doi: 10.1158/2767-9764.CRC-22-0477 (PMC10103717; doi:10.1158/2767-9764.CRC-22-0477)
Supplement: Figure S4 — Tumor experiments showing monotherapy testing of IOA-244 [file crc-22-0477-s04.pdf]

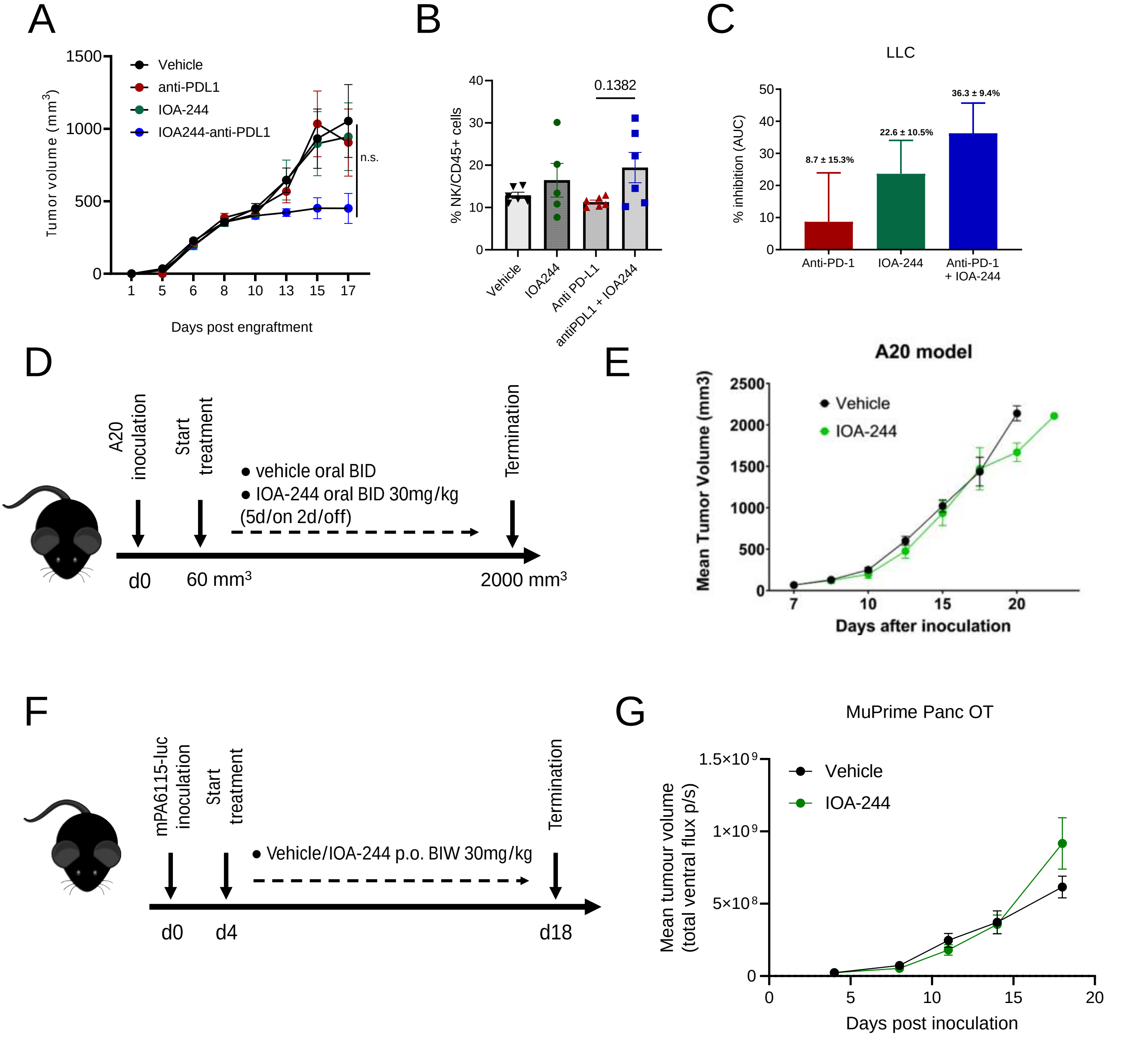

Supplementary figure 4: (A) Tumor volume of CT26 colorectal mouse model, upon treatment with vehicle, anti-PDL1, IOA-244/anti-PDL1. (B) Flow cytometry analysis from CT26 tumors showing quantification of NK cells out of the CD45+cells. (C) AUC (area under the curve) of LLC tumor model showed in Figure 4F. (D-E) Experimental layout and tumor volume of A20 model. (F-G) Experimental layout and tumor volume of MuPrime (KPC-derived) orthotopic pancreatic model, as assessed by caliper measurement.
