# Supplementary material for: IOA-244 is a Non–ATP-competitive, Highly Selective, Tolerable PI3K Delta Inhibitor That Targets Solid Tumors and Breaks Immune Tolerance
Source: Cancer Res Commun. 2023 Apr 14;3(4):576–91. doi: 10.1158/2767-9764.CRC-22-0477 (PMC10103717; doi:10.1158/2767-9764.CRC-22-0477)
Supplement: Table S1 — Table summarizing the IC50 values derived from previous published experiments performed with IOA-244 [file crc-22-0477-s05.docx]

Table S1: Summary of main findings from Haselmayer et al., 2014.

| **Assay** | **Readout** | **IC50** |
| --- | --- | --- |
| Ramos cells stimulated with IgM (15’) | Intracellular pAKT | 280 nM |
| Human PBMC stimulated with IgM (4days) | Cell proliferation (gated on CD19+) | 61 nM |
| Human PBMC stimulated with IgM (5’) | Intracellular pAKT (gated on CD19+) | 1550 nM |
| Human PBMC stimulated with IgM (18h) | CD69 upregulation (gated on CD19+) | 307 nM |
| Murine blood stimulated with IgD | Intracellular pAKT (gated on B220+) | 463 nM |
| Murine blood stimulated with IgD | CD69 upregulation (gated on B220+) | 443 nM |
| Ex vivo assay, upon *in vivo* treatment with MSC2360844 (1, 3, 10 mg/kg) | - Intracellular pAKT (gated on B220+) | - ED50=3 mg/kg at 1h - ED50=10 mg/kg at 2h - NS at 4h and 6h |

**Table S1 legend:**

Table summarizing the IC50 measurements of IOA-244/MSC2360844, by performing the listed assays and experimental redouts. (Data are extracted from Haselmayer et al., Frontiers in Immunology, 2014).

.
