# Supplementary material for: IOA-244 is a Non–ATP-competitive, Highly Selective, Tolerable PI3K Delta Inhibitor That Targets Solid Tumors and Breaks Immune Tolerance
Source: Cancer Res Commun. 2023 Apr 14;3(4):576–91. doi: 10.1158/2767-9764.CRC-22-0477 (PMC10103717; doi:10.1158/2767-9764.CRC-22-0477)
Supplement: Table S2 — Table showing values of microsomal stability of IOA-244 [file crc-22-0477-s06.docx]

| **Compound** | **Compound remaining (% of 0 min)** | | | | | | **Metabolic stability** | | | |
| --- | --- | --- | --- | --- | --- | --- | --- | --- | --- | --- |
|  | 0 min | 5 min | 15 min | 30 min | 45 min | Control | CL_int_ (µL/min/mg protein) | SE CL_int_ | T_1/2_ (min) | n |
| Umbralisib | 100 | 114 | 108 | 101 | 111 | 109 | -0.912 | 3.55 | -1520 | 5 |
| IOA-244 | 100 | 91.4 | 82.2 | 84.4 | 83.9 | 94.0 | 6.41 | 3.48 | 216 | 5 |

Table S2: Metabolic stability of umbralisib and IOA-244 in human liver microsomes

CL_int_ = intrinsic clearance; SE = standard error.

**Table S2 legend:**

Table showing time-dependent depletion of umbralisib and IOA-244 by NADPH-supplemented human liver microsomes. The data are expressed as a percentage of the compound remaining at each time compared to time 0 min, and represent the mean ± SE
