# Supplementary material for: IOA-244 is a Non–ATP-competitive, Highly Selective, Tolerable PI3K Delta Inhibitor That Targets Solid Tumors and Breaks Immune Tolerance
Source: Cancer Res Commun. 2023 Apr 14;3(4):576–91. doi: 10.1158/2767-9764.CRC-22-0477 (PMC10103717; doi:10.1158/2767-9764.CRC-22-0477)
Supplement: Table S3 — Table showing the raw data of the AUC value of figure 2A [file crc-22-0477-s07.docx]

**Supplementary Table 3. AUC values obtained in 67 lymphoma cell lines exposed to IOA-244 for 72 hours.**

| **cell line name** | **Histology** | **IOA-244 AUC** |
| --- | --- | --- |
| SP49 | MCL | 262407 |
| FARAGE | GCB DLBL | 468617 |
| PCL12 | CLL | 487228 |
| OCILY3 | ABC DLBCL | 497334 |
| GRANTA519 | MCL | 505452 |
| SUDHL6 | GCB DLBL | 506725 |
| HG3 | CLL | 509827 |
| SP53 | MCL | 521984 |
| OCILY7 | GCB DLBL | 532680 |
| HH | CTCL | 552541 |
| KARPAS1106P | PMBCL | 584733 |
| REC1 | MCL | 585057 |
| WSUDLCL2 | GCB DLBL | 586670 |
| MEC1 | CLL | 589944 |
| SUDHL8 | GCB DLBL | 597506 |
| TMD8 | ABC DLBCL | 597878 |
| HAIRM | MZL | 618678 |
| JVM2 | MCL | 619587 |
| RI1 | ABC DLBCL | 633578 |
| A20 | murine B cell lymphoma | 640045 |
| SUDHL5 | GCB DLBL | 641724 |
| OCILY8 | GCB DLBL | 662301 |
| HC-1 | MZL | 670133 |
| MJ | CTCL | 691005 |
| MINO | MCL | 712275 |
| SUDHL4 | GCB DLBL | 712926 |
| NK92 | NKL | 720107 |
| PFEIFFER | GCB DLBL | 724573 |
| YT | NKL | 725468 |
| ESKOL | MZL | 751939 |
| OCILY18 | GCB DLBL | 754191 |
| TOLEDO | GCB DLBL | 797347 |
| JEKO1 | MCL | 799763 |
| HBL1 | ABC DLBCL | 807810 |
| DOHH2 | GCB DLBL | 809436 |
| KARPAS422 | GCB DLBL | 823715 |
| VL51 | SMZL | 834318 |
| DERL7 | NKL | 841819 |
| KARPAS1718 | SMZL | 870133 |
| MAVER1 | MCL | 875766 |
| VAL | GCB DLBL | 883204 |
| OCILY19 | GCB DLBL | 901185 |
| SSK41 | SMZL | 914469 |
| DB | GCB DLBL | 926887 |
| AM-HLH | HL | 932290 |
| SUDHL16 | GCB DLBL | 933863 |
| KIJK | ALCL, ALK+ | 946278 |
| HUT78 | SS | 951755 |
| SUDHL2 | ABC DLBCL | 953122 |
| SUDHL1 | ALCL, ALK+ | 956008 |
| RCK8 | ABC DLBCL | 966387 |
| KM-H2 | HL | 968136 |
| Z138 | MCL | 974145 |
| UPN1 | MCL | 979416 |
| OCILY1 | GCB DLBL | 984827 |
| OCILY10 | ABC DLBCL | 994583 |
| U2932 | ABC DLBCL | 1017183 |
| MAC1 | cALCL, ALK- | 1026194 |
| L-1236 | HL | 1030294 |
| H9 | SS | 1066697 |
| KHYG | NKL | 1069672 |
| SUDHL10 | GCB DLBL | 1080674 |
| L82 | ALCL, ALK+ | 1111835 |
| L428 | HL | 1135365 |
| KARPAS299 | ALCL, ALK+ | 1146572 |
| NKYS | NKL | 1149797 |
| FEPD | PTCL-NOS | 1151149 |

ABC DLBCL, Activated B-cell-like diffuse large B-cell lymphoma; ALCL, anaplastic large cell lymphoma; CTCL, cutaneous T cell lymphoma; CLL, Chronic lymphocytic leukemia; GCB DLBCL, Germinal center B-cell-like diffuse large B-cell lymphoma; HL, Hodgkin lymphoma; MCL, Mantle cell lymphoma; MZL, Marginal zone lymphoma; NKL, NK cell lymphoma; PMBCL, Primary mediastinal B-cell lymphoma; PTCL-NOS, peripheral T-cell lymphoma-not otherwise specified; SS, Sezary syndrome.
